# Supplementary material for: Prediction of Chronological Age in Healthy Elderly Subjects with Machine Learning from MRI Brain Segmentation and Cortical Parcellation
Source: Brain Sci. 2022 Apr 29;12(5):579. doi: 10.3390/brainsci12050579 (PMC9139275; doi:10.3390/brainsci12050579)
Supplement: Supplementary file 1 [file brainsci-12-00579-s001.zip › brainsci-1668206-supplementary.pdf]

Supplementary Material

Support Vector Machines are maximum-margin classifiers. They maximize the distance between the closest vectors of each class. SVMs can also be used for regression (SVR). SVR presents minor differences with SVM for classification, namely SVR uses a margin of tolerance or epsilon otherwise predicting the output which is a real number would be cumbersome. However, SVR follows the same principle as in SVC, error minimization by individualizing the hyperplane that minimizes the margin while assuring that the margin of error is within reach.

We implement a linear support vector regressor using the scikit-learn 1.0.2 which is the same library used for the PLS and XGBoost models described in the manuscript. The implementation of SVR is based on the liblinear library rather than libsvm, a popular open-source machine learning libraries written in C++ implementing the Sequential minimal optimization algorithm for kernelized support vector machines. The liblinear is indicated for large datasets allowing for better scalability and flexibility in the choice of penalties and loss functions.

Prior to building and fitting the model, feature scaling is performed using the StandardScale() function declared in the sklearn library. Next, we fit the Linear Support Vector Regression Model to the training dataset, having previously left aside the 25% of the original dataset for testing.
We perform Grid Search Cross Validation using 3 folds for each of 9 candidates defined in the hyperparameters search space ('C':[1.0, 10,100],'epsilon':[0.0, 0.1, 1.0]), totalling 27 fits. The performance of the SVR estimator on the dataset is shown in the below table with the best free parameters for regularization (C) and error tolerance (Epsilon).

SVR	C	Epsilon	MAE	MXE	MAPE	MEDAE	R^2
	1.0	0.1	2.5449	10.7894	0.0331	2.0528	0.3364
